# Optimization, Characterization, and Anticancer Potential of Silver Nanoparticles Biosynthesized Using *Olea europaea*

**DOI:** 10.1155/2022/6859637

**Published:** 2022-09-26

**Authors:** Afnan I. Felimban, Njud S. Alharbi, Nehad S. Alsubhi

**Affiliations:** ^1^Department of Biological Sciences, Faculty of Science, King Abdulaziz University, Jeddah 21589, Saudi Arabia; ^2^Department of Biological Science, Faculty of Science, University of Jeddah, Jeddah, Saudi Arabia

## Abstract

Green synthesis has attracted significant attention as an eco-friendly, low-cost, energy-efficient, and non-toxic method for preparing silver nanoparticles (AgNPs) for cancer therapy. This study optimized the green synthesis of AgNPs using *Olea europaea* extracts and evaluated their anticancer potential. The biosynthesized AgNPs were characterized using various methods, showing stable AgNPs with a desirable morphology and high yield, improving the properties of AgNPs for various medicinal applications. The biosynthesized AgNPs were predominantly spherical, with small sizes ranging from 13 to 21 nm and highly stable at −23 and −24 mV. The findings of this study suggest that green-synthesized AgNPs using *Olea europaea* and sunlight possess significant anticancer activity against cancer cells *in vitro*. Further investigation of green synthesis would help to form high-quality AgNPs that have promising potential in treating disease and fighting undesirable pathogens.

## 1. Introduction

Nanotechnology is a scientific field that involves the creation and application of nanosized particles. Nanoparticles are well-known as particles that range between 1 nm and 100 nm in diameter. The physical, chemical, and biological properties of these particles can be easily modified and used in various applications [1]. Therefore, they have attracted intensive studies in different fields, such as biology, chemistry, agriculture, and electronics [2]. Consequently, these small particles have a positive impact on food production, pharmaceuticals, and drug and gene delivery [3, 4].

Nanoparticles can be classified into several types based on their morphology and physical and chemical properties. Metal nanoparticles such as gold, silver, zinc, and iron can be made of pure metals or compounds. Among the metal nanoparticle types, silver nanoparticles (AgNPs) are the most important. AgNPs have numerous applications in different fields because of their unique properties [5–7].

Top-down and bottom-up methods are the two major approaches for synthesizing nanoparticles [8]. The top-down approach involves breaking down bulk material structures into nanosized particles. Top-down methods offer high controllability of pattern size and shape while typically incurring high costs. In contrast, the bottom-up approach involves building nanomaterials from smaller entities. Bottom-up methods offer advantages such as low cost and productivity, while the lack of control over precision in particle shape and size is their biggest drawback. Sol-Gel and green synthesis are considered bottom-up methods [9].

Green synthesis is an easy way to obtain nanoparticles with more advantages than disadvantages. Green synthesis provides a fast, economical, nontoxic, eco-friendly, and biocompatible alternative to physical and chemical techniques [10]. The synthesis of AgNPs from plants offers many advantages such as easy processing, cost efficiency, and no culture requirement [11]. Phytochemicals in plants, such as polyphenols, flavonoids, tannins, proteins, and sugars, are responsible for synthesizing nanoparticles and serve as reducing and stabilizing agents. In addition to their powerful reduction properties, they can reduce Ag ^+^ into Ag^0^. Furthermore, they serve as capping agents for the coating of nanoparticles, providing greater stability in the size and shape of nanoparticles [12].

Reaction conditions, such as extract concentration, temperature, pH, and light intensity, can influence the production, characterization, and application of AgNPs [13]. Optimization of the reaction conditions can help produce nanoparticles with well-defined sizes and morphologies, thereby improving their properties [14, 15].

The present study optimized the green synthesis of AgNPs using *Olea europaea* extracts for improved biological applications. The fundamental aim of the study was to provide an alternative to physical and chemical techniques for producing high-quality AgNPs that would improve their biological efficacy without introducing hazardous chemicals. The aim included examining the antitumor activities of the biosynthesized AgNPs against breast cancer cells, achieving remarkable results.

## 2. Materials and Methods

**Figure 1:**
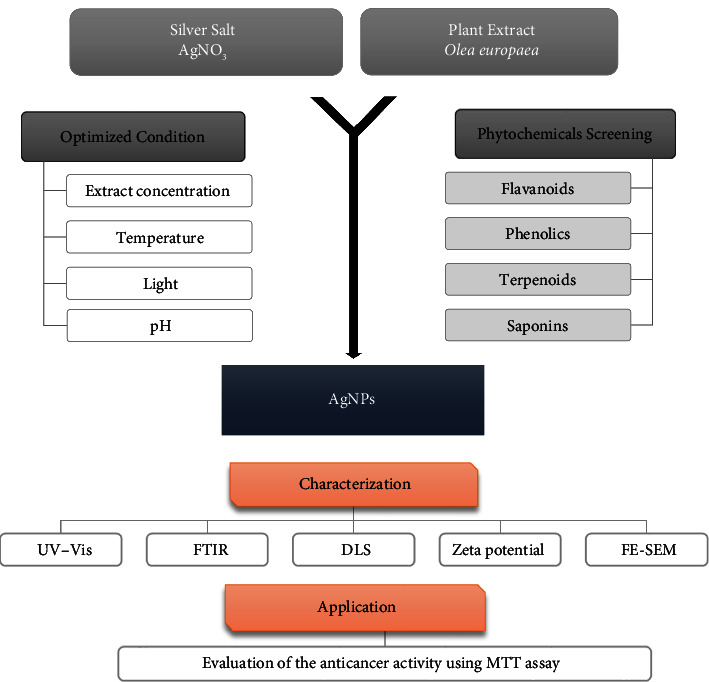
Schematic diagram of the green synthesis of silver nanoparticles (AgNPs) using *Olea europaea* extracts: phytochemical screening, optimization, characterization, and anticancer activity evaluation.

The green synthesis of AgNPs using *O. europaea* extracts along with phytochemical screening, optimization, characterization, and anticancer activity evaluation is illustrated in the schematic diagram (Figure 1).

### 2.1. Plant Collection and Aqueous Extracts Preparation

**Figure 2:**
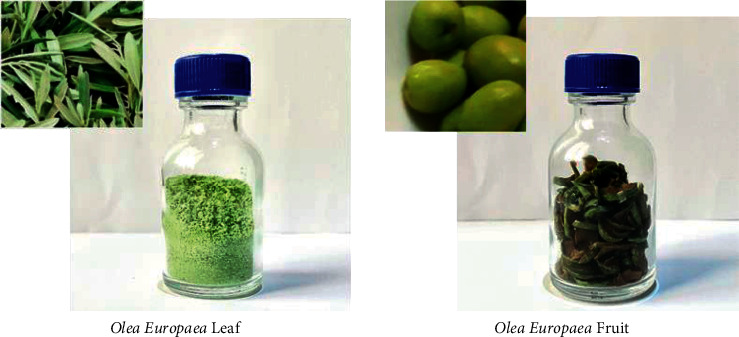
*Olea europaea* leaves and fruit.

Fresh *O. europaea* plants (Figure 2) were collected from the Al-Jouf region, Saudi Arabia, by the Al-Jouf Agricultural Development Company. Aqueous extracts were determined following the method described in a previous study [16]. The collected plant materials were washed individually with tap water and then washed several times with distilled water to remove contaminants. The washed plant materials were left to dry completely in the shade at 24°C. The dried leaves were then grounded into a fine powder, and the fruit slices were stored in airtight containers. For aqueous extraction, five grams of the fine powder were mixed with deionized water, and the mixture was boiled and then filtered through a coffee filter and Whatman No. 1 filter paper. The extracts were stored in a bottle at −4°C for further use.

### 2.2. Phytochemical Screening

The phytochemicals in the *O. europaea* extracts were identified using the standard procedures described in Table 1. These are as reported in previous studies, [17–19]. Phytochemicals are involved in the reduction and coating of AgNPs.

### 2.3. Optimization of Green Synthesis of AgNPs

Processing parameters, such as extract concentration, temperature, pH, and light intensity, were optimized to improve the quality of the AgNPs. The optimum AgNPs conditions were determined using the parameters with the best results to obtain the best possible production and quality of AgNPs. The primary synthesis of AgNPs was performed following the procedure described in a previous study [24]. An amount of aqueous plant solution was added to 1 mM of AgNO_3_ solution at a specific ratio, and the green synthesis was monitored at various time intervals. The reduction of the silver ions into AgNPs was followed by a colour change of the solution from light to reddish-brown depending on the parameters studied, the change in colour confirmed the reduction. It is well-known that AgNPs exhibit a dark brownish colour in an aqueous solution owing to the excitation of the surface plasmon vibrations in the metal nanoparticles [25, 26]. Also, the results indicated that the phytochemicals of O. europaea extracts successfully reduced Ag + to silver nanoparticles in an aqueous solution. Under direct sunlight, the reaction led to rapid nanoparticle formation, possibly because the photons from sunlight accelerated the reaction [27]. It was also reported that a long-lasting dark brown colour confirmed that all silver ion has completely reduced into AgNPs [28]. The AgNPs solutions were then centrifuged at 10,000 RPM for 10 min; the centrifugation process was repeated three times. Finally, the purified AgNPs were collected to analyse their characteristics.

### 2.4. Characterization Techniques

The biosynthesized AgNPs were characterized using ultraviolet-visible spectroscopy (UV-Vis), Fourier-transform infrared (FTIR) spectroscopy, dynamic light scattering (DLS), zeta potential analysis, and field-emission scanning electron microscopy (FE-SEM). For illustration, the green synthesis of AgNPs was monitored using a Cary® 50 ultraviolet-visible spectrophotometer in the 300–600 nm wavelength range. The association of biomolecules involved in the formation and stabilization of AgNPs were detected using an FTIR spectrometer (Fisher Nicolet iS10) in the 600–4000 cm^−1^ wavelength range. The particle hydrodynamic size distribution and stability of the AgNPs were observed using DLS and zeta potential using a Zetasizer Nano (Malvern Instruments Ltd., U.K.). The morphology, size, and shape of biosynthesized AgNPs are considered essential characteristics of nanoparticle systems because they determine their biological activity and toxicity [29]. Thus, they were analysed using field-emission scanning electron microscopy (JEO, JSM 7600F).

### 2.5. Cell Lines and Cell Cultures

The human hormone-dependent breast cancer cell lines, MCF7 and T47D, were obtained from the King Fahd Medical Research Centre. The cells were maintained in a high-glucose Dulbecco's Modified Eagle Medium (DMEM), supplemented with 10% fetal bovine serum (FBS) and 1% penicillin-streptomycin (Beijing Solarbio Science and Technology Co., Ltd., China). Cancer cells were grown in a humidified 5% CO_2_ incubator at 37°C and passaged three times per week.

### 2.6. *In Vitro* Assay for Cytotoxicity Activity (MTT Assay) Anticancer Activity

The cytotoxicity of green-synthesized AgNPs dissolved in 0.3 dimethyl sulfoxide DMSO (AgNPs), *O. europaea* leaf extract (OLE) and AgNPs dissolved in leaf extract (AgNPs-OLE) samples were evaluated using the MTT assay. An MTT assay was performed according to the method described in a previous study [30]. Cells were seeded at a concentration of 1 × 10^4^ cells per well in 96 well plates supplied with DMEM. The plates were incubated for 24 h at 37°C and maintained in a 5% CO^2^ atmosphere to allow the cells to adhere overnight. Thereafter, the cells were treated with different concentrations (25, 50, 100, 150, and 200 *μ*g/mL) of treatments in triplicate and incubated for 24 h, and the control cell cultures were left untreated. After incubation, the old media was discarded, and 100 *μ*L of MTT solution was added to each well. The 96 well plates were then incubated for approximately 3–4 h until formazan crystals formed. The medium was then replaced with a 100 *μ*L of DMSO solution and the OD was measured using an ELISA reader at 570 nm. The cytotoxic effects of the three treatments on breast cancer cells were expressed as the % cell viability using the following formula:

(1) $$\begin{array}{c} \%\text{cell viability}=\frac{\text{Absorbance treated cells}-\text{Absorbance blank}}{\text{Absorbance control cells}-\text{Absorbance blank}}\times 100. \end{array}$$

- Blank refers to the background, which means the medium, MTT solution, and DMSO.
- Control cells refer to untreated cells.

### 2.7. Statistical Analyses

Statistical analyses were performed using SPSS software. All data were analysed using one-way analysis of variance (ANOVA) followed by Tukey's post hoc test for multiple comparisons, and significance was considered *P* < 0.05.

## 3. Results and Discussion

### 3.1. Phytochemical Screening Analysis

Phytochemical screening analysis confirmed the presence of flavonoids, phenols, triterpenes, and saponins in the *O. europaea* extracts, as listed in Table 2. These phytochemicals play an essential role in the bio-reduction of Ag ^+^ to Ag^0^ and the stabilization of biosynthesized AgNPs.

### 3.2. Optimization of the Green Synthesis Using UV-Vis Spectroscopy Analysis

#### 3.2.1. Effect of Extract Concentration

**Figure 3:**
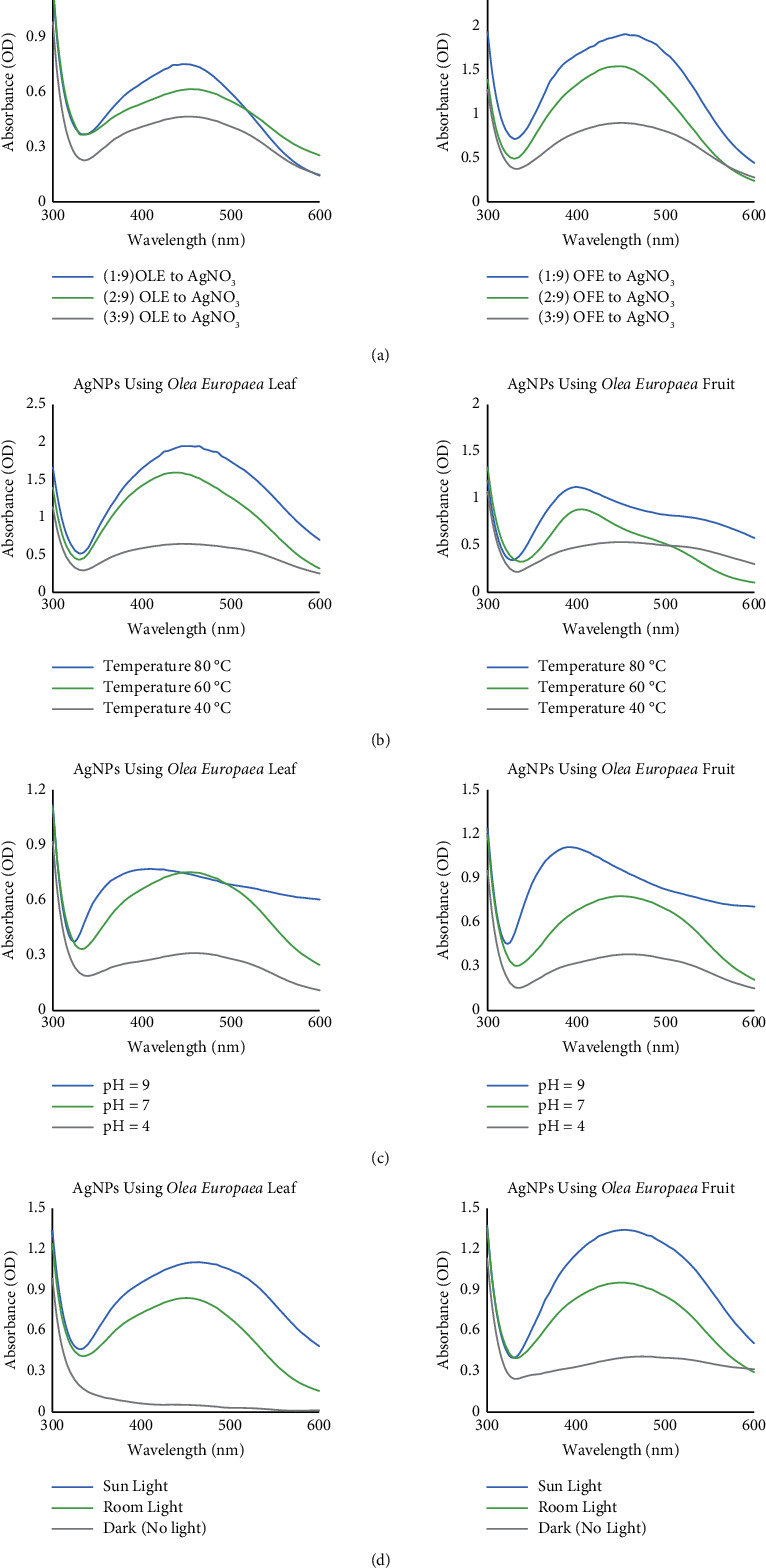
UV-vis absorption spectra of AgNPs synthesized using *Olea europaea* extracts at various operational parameters: (a) extract concentration using different ratios of plant extract to AgNO_3_, (b) temperature, (c) change in pH, and (d) change in light intensities.

To examine the effect of the extract concentration, three different volumes of 1, 2, and 3 mL of each extract were added separately to 9 mL of 1 mM AgNO_3_ solution. Thus, reaction mixtures with the ratios of 1 : 9, 2 : 9, and 3 : 9 plant extract to 1 mM AgNO_3_ silver nitrate were prepared, following the process described in an earlier study [31]. Increasing the plant extract volume in the reaction mixture increased the productivity of AgNPs, as shown in Figure 3(a). The plasmon resonance band in the 3 : 9 ratio sample was higher and appeared at a wavelength of 449 nm for the olive leaf and 464 nm for the olive fruit extract. Increasing the extract concentration increases the biomolecule content, resulting in a more intense colour. This finding is consistent with that reported in previous research [32]. Biomolecules act as reducing and capping agents at higher extract concentrations, protecting the synthesized nanoparticles from aggregation and affecting their size.

#### 3.2.2. Effect of Temperature

The biosynthesized AgNPs were prepared at different reaction temperatures (40, 60, and 80°C), following the method of a previous study [33]. The results show an increase in the formation rate of AgNPs with increasing reaction temperature, as illustrated in Figure 3(b). The results also showed that the UV spectral wavelength decreased as the temperature increased, reducing the size of the AgNPs due to the rapid consumption of reactants. Higher temperatures resulted in higher rates of AgNPs formation. This is attributed to the fact that silver ions are consumed faster, leaving fewer opportunities for nanoparticles to grow. This finding is consistent with findings in an earlier study [34], confirming that increasing the temperature can increase the absorbance intensity at which small-sized AgNPs form rapidly. Another study [35] found the same results, confirming that increasing temperature can rapidly form AgNPs, directly affecting the nanoparticle size and shape.

#### 3.2.3. Effect of pH

**Figure 4:**
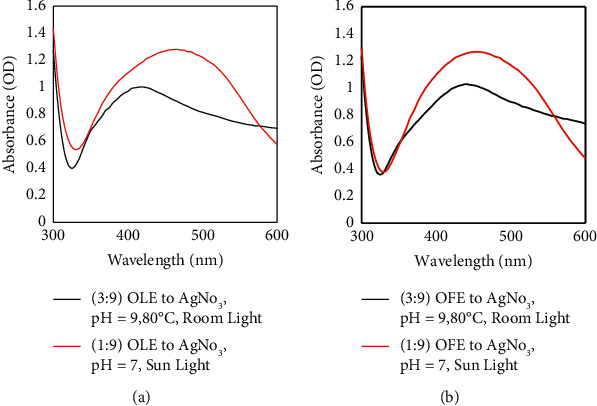
UV-vis absorption spectra of AgNPs synthesized using *Olea europaea* (a) leaf extract and (b) fruit extract at optimum production conditions in the current study for green synthesis of AgNPs.

The effect of pH on the synthesis process was investigated at three different pH values: acidic (pH = 4), neutral (pH = 7), and basic (pH = 9). The pH of the green synthesis mixture was adjusted to the desired value using 0.1 N sodium hydroxide and 0.5 N acetic acid.  Figure 4(c) shows the UV-vis absorption spectra of the AgNPs synthesized at three different pH values. The results showed that the highest reaction rate was observed at a pH of 9. The results show that an alkaline pH can promote the reaction of synthesized AgNPs, confirming the results reported in a previous investigation [36]. These results suggest that the efficient production of small AgNPs could be due to a large number of functional groups available for Ag binding under higher pH conditions, as earlier reported [37]. For illustration, the primary influence of the reaction pH is its ability to change the electrical charges of biomolecules, which may affect their capping and stabilizing properties and, subsequently, the growth of the synthesized AgNPs [38]. The alkaline pH allows more hydroxyl (O-H) groups of plant extracts to participate in the reduction reaction, improving the yield of the green synthesis [39]. The results, therefore, showed that the nanoparticle size decreased with the increasing pH with lower aggregation, thus increasing particle stability. Similar findings have been reported in a previous investigation. A previous study reported that the AgNPs synthesis rate increased as the pH increased to 7 and then decreased when the pH value increased further [40]. In contrast, the reduction was mainly accomplished at acidic pH through ionic bonding and biomolecules due to the positively charged functional groups. Thus, many biomolecules bind when synthesizing AgNPs, resulting in agglomeration and larger-sized AgNPs [41, 42]. Hence, adjusting the pH could help control the size and stability of the AgNPs.

#### 3.2.4. Effect of Light

Light can considerably influence the green synthesis of AgNPs [43]. A spectral analysis of the samples grown under dark, room light, and sunlight conditions was performed using a UV-vis spectrophotometer [44]. The results in Figure 4(d) show the significant impact of light intensity on the absorbance patterns. Sunlight can help achieve the best formation rate of AgNPs. Under the sunlight condition, the plasmon absorption band was the highest. The UV-vis spectra showed maximum absorbance at a wavelength of 465 nm for olive leaf and 455 nm for olive fruit extract. However, no formation of AgNPs was observed under dark conditions after 24 h, and an insubstantial amount of AgNPs was observed when olive fruit extract was used. These results suggest an increase in absorbance when the light intensity increases. A possible explanation might be that a more significant number of photons in direct sunlight can help catalyse the reducing process, significantly promoting the green synthesis of AgNPs, as previously reported [45].

**Figure 5:**
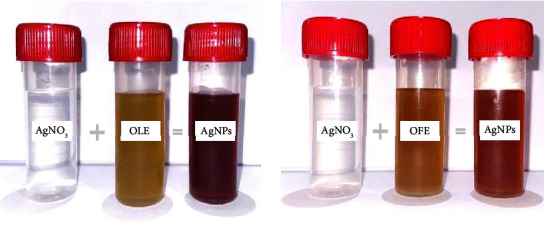
Visual observation of the green synthesis of silver nanoparticles (AgNPs) using *Olea europaea* leaf extract (OLE), and *Olea europaea* fruit extract (OFE).

After studying these parameters, two sets of working conditions were selected as the optimum reaction conditions to produce high-quality AgNPs. By examining the two sets, the set of 3 : 9 plant extract to AgNO_3_, pH value of 9, temperature of 80°C, and room light produced a lower amount of AgNPs compared with 1 : 9 plant extract to AgNO_3_, pH value of 7, and sunlight, as shown in Figure 4. Thus, the optimum conditions in the current study for the valuable, simple, and rapid green synthesis of AgNPs were 1 : 9 plant extract to AgNO_3_, a pH value of 7, and sunlight, as shown in Figure 5. With the selected optimum production conditions, small AgNPs formed in a large amount within a few minutes, and they were simultaneously characterized by high stability. For example, using sunlight in the green synthesis of AgNPs can speed up the synthesis and still yield a high concentration of AgNPs characterized by good stability, as recorded in a previous investigation [46]. This result agrees with many previous studies that have reported an effective green synthesis of AgNPs using sunlight [47–49].

### 3.3. FTIR Analysis

**Figure 6:**
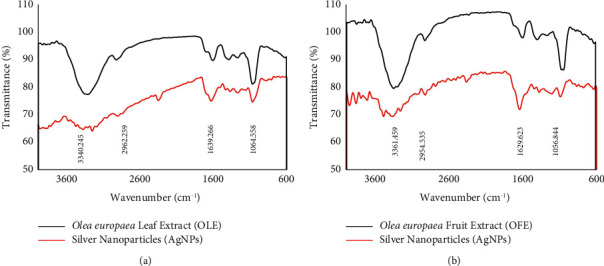
FTIR spectrum of plant extracts and AgNPs synthesized using *Olea europaea* (a) leaf extract and (b) fruit extract.

An FTIR analysis helped identify the various biomolecules of the plant extracts responsible for capping and efficiently stabilizing the AgNPs.  Figure 6(a) shows the FTIR absorption bands of *O. europaea* leaf extracts and biosynthesized AgNPs. The results showed a broad and robust peak at 3340.2 cm^−1^, representing the hydroxyl (O-H) functional group and indicating the presence of a phenolic group. Additionally, the absorption peaks that emerged at 2962.2 and 1639.2 cm^−1^ correspond to the functional groups (C-H) and (*C* = *C*), respectively. In addition, the spectrum observed at 1064.5 cm^−1^ points to (C-O).  Figure 6(b) displays several absorption peaks in the IR spectrum of *O. europaea* fruit extract and biosynthesized AgNPs. A broad peak, responsible for (O-H) stretching was seen at 3361.4 cm^−1^. Characteristic peaks were also observed at 2954.5 (C-H), 1629.6 (*C* = *C*), and 1056.8 (C-O) cm^−1^. The FTIR spectrum results confirmed that the bioactive compounds present in the plant extracts were adsorbed on the surface of the biosynthesis of AgNPs, which is in agreement with that reported in an earlier study [50]. The bio-compounds found in the tested *O. europaea* parts, phenolics, and flavonoids played an essential role in reducing, capping, and stabilizing the green-synthesized AgNPs, thus enhancing their properties [51, 52].

### 3.4. DLS and Zeta Potential

**Figure 7:**
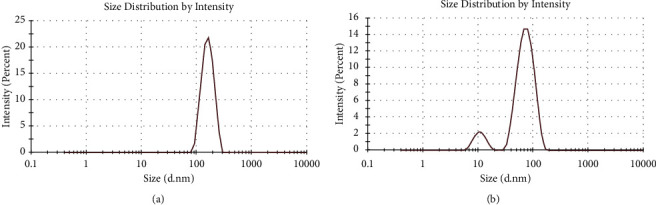
Particle size distribution of biosynthesized AgNPs using *Olea europaea* (a) leaf extract and (b) fruit extract.

**Figure 8:**
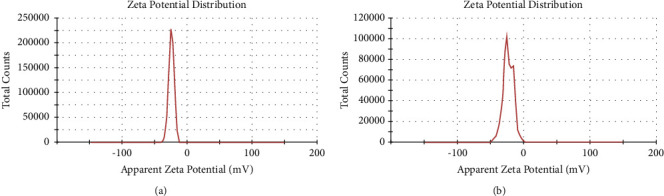
Zeta potential measurement of biosynthesized AgNPs using *Olea europaea* (a) leaf extract and (b) fruit extract.

From the DLS analysis (Figure 7), the average hydrodynamic size of synthesized AgNPs using *O. europaea* leaf extract was found to be 182 nm with a single peak and a polydispersity index (PDI) of 0.2. In addition, the zeta potential was −24 mV with a sharp peak. However, the average hydrodynamic size, PDI, and zeta potential for synthesized AgNPs using *O. europaea* fruit extract were 66 nm, 0.2, and −23 mV, respectively. These numerical values for PDI are generally acceptable because the smaller the PDI, the more homogeneous nanoparticles are produced [37]. Zeta potentials of −23 and −24 mV (Figure 8) indicated a stable dispersion without notable AgNPs agglomeration over an extended time in the solution. Previous investigations have stated that nanoparticles with charges in the range of +30 mV to −30 mV are very stable, and those in the range of +15 mV to−25 mV are moderately stable [53–55].

### 3.5. FE-SEM Analysis

**Figure 9:**
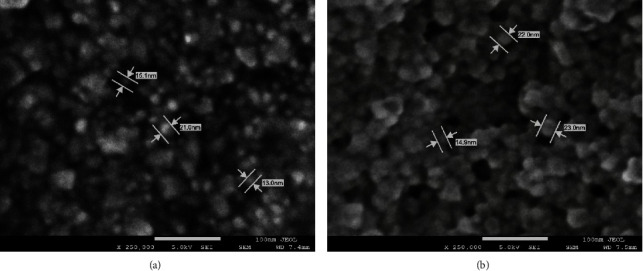
FE-SEM images of biosynthesized AgNPs using *Olea europaea* (a) leaf extract and (b) fruit extract.

FE-SEM images helped visualize the morphology, shape, and size of the biosynthesized AgNPs.  Figure 9 shows micrograph images of the biosynthesized AgNPs using O. europaea extracts. The FE-SEM revealed that the nanoparticles were predominantly spherical, with some aggregation. However, an agglomeration might be observed due to the high concentration during manual sample preparations. The size of the AgNPs varied between 13–21 nm for O. europaea leaves and between 14.9–23 nm for O. europaea fruit. Similar results were previously reported [56]. The size variation between the AgNPs synthesized using O. europaea leaves and fruit is due to the difference in the molecular make-up of the plant cell. The type and quantity of biomolecules and secondary metabolites in the cell affect the size and surface properties of the synthesized nanoparticles [57]. For illustration, the phytochemical components of the plant extract act as reducing and coating agents, influencing the size and stabilization of the nanoparticles. The robust coating of the synthesized nanoparticles can offer more stability, protecting against agglomeration and aggregation [58, 59].

After characterization of biosynthesized AgNPs, a better anticancer application would be obtained for AgNPs biosynthesized using *O. europaea* leaf rather than fruit because of their better stability, smaller size, and good shape.

### 3.6. Anticancer Activity Evaluation

**Figure 10:**
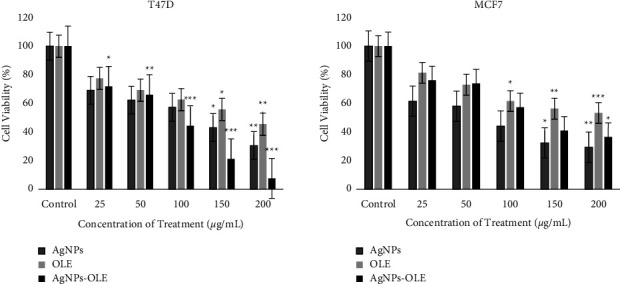
Cytotoxicity effects of AgNPs, OLE, and AgNPs-OLE on the viability of T47D and MCF7 after 24 h exposure. (Results are viable in comparison with the control (untreated cells). Data were analysed by applying one-way analysis of variance (ANOVA) with Tukey's posthoc tests; ^*∗*^*P* value <0.05, ^*∗∗*^*P* value <0.01, and ^*∗∗∗*^*P* value <0.001).

The anticancer activities of AgNPs, OLE, and AgNPs-OLE were evaluated *in vitro* against T47D and MCF7 breast cancer cell lines, and their IC50 values were determined from a graph of cell viability measured over a range of concentrations between 25 and 200 *μ*g/mL. The results showed that AgNPs, OLE, and AgNPs-OLE effectively inhibited the proliferation of T47D and MCF7 cancer cells in a dose-dependent manner after 24 h of treatment (Figure 10). The percentage of cell death in T47D and MCF7 cells gradually increased with the increasing concentrations of the three treatments. The cytotoxicity of the three treatments also revealed that their higher concentrations had significantly more toxic effects on the cell viability of both T47D and MCF7, suggesting that the AgNPs, OLE, and AgNPs-OLE mediated cell death in a concentration-dependent manner.

The IC50 values were 116 *μ*g/mL for T47D cells in the presence of AgNPs, 176 *μ*g/mL in the presence of OLE, and 84 *μ*g/mL in the presence of AgNPs-OLE. The IC50 values obtained were less than 200 *μ*g/mL. In contrast, the IC50 values of AgNPs, OLE, and AgNPs-OLE on MCF7 cells were 80, 200, and 132 *μ*g/mL, respectively. Thus, the results clearly demonstrated that the three treatments notably inhibited the cancer cells at moderate concentrations. Overall, the results of this study suggest that green-synthesized AgNPs possess significant anticancer activity in cancer cell lines.

The higher efficacy of the synthesized AgNPs using O. europaea leaf extract against breast cancer cells might be due to their smaller size, which facilitates cell entry. The phytochemicals of O. europaea leaf extract enhanced the AgNPs biocompatibility and facilitated cellular access. A possible explanation for the results is that AgNPs perform as cancer therapeutics due to their potential ability to disrupt the mitochondrial respiratory chain, which induces the production of reactive oxygen species, causing DNA damage [60, 61].

Although a few previous studies considered the biosynthesis of AgNPs using O. europaea extracts [38, 40, 52], promoting AgNPs production using sunlight was not considered. The current study evaluated the effect of light intensity on the biosynthesized AgNPs using O. europaea, and a quick bio-reduction and small stable AgNPs with high yield were achieved using sunlight. We believe our study makes a significant contribution to the literature because, to our knowledge, this is the first inclusive study to apply green-synthesized AgNPs using O. europaea extract and sunlight to MCF7 and T47D cells. The findings presented provide convincing evidence of the promising biomedical and therapeutic applicability of AgNPs green-synthesized using O. europaea, which warrants further investigation and is clinically and academically relevant. However, this study had some limitations that need to be considered. With the variety and complexity present in the phytochemical composition of plant extracts, it is challenging to determine their full role in the reduction reactions and coating processes that control the size, shape, and stability of the nanoparticles [62]. Further, the type of solvent used is critical in the green synthesis of nanoparticles. The universal solvent, water, was chosen in this study as it is the safest, least expensive, and most eco-friendly solvent. Moreover, factors such as agglomeration and aggregation should be studied extensively, as they influence the efficiency of AgNPs applications [63, 64]. Another limitation of this study is that only in vitro experiments were conducted, and in vivo experiments would be necessary for the future clinical translation of the results.

## 4. Conclusion

Green synthesis of AgNPs using *O. europaea* extracts was performed, and AgNPs with well-defined morphologies were created. The results showed that the reaction parameters had substantial effects on the green synthesis of the AgNPs. Based on the results of the current study, the valuable, simple, and rapid formation of green-synthesized AgNPs can be achieved using sunlight. The FTIR analysis revealed the presence of some functional groups (O-H), (C-H), (*C* = *C*), and (C-O) and confirmed that the bio-compounds, phenolics, and flavonoids present in the plant extracts were adsorbed on the surface of the AgNPs, thus enhancing their properties and future applications. The different biosynthesized AgNPs were spherical and ranged in size between 13–21 nm for *O. europaea* leaf and between 14.9–23 nm for *O. europaea* fruit. Furthermore, the cytotoxicity results of green-synthesized AgNPs using *O. europaea* leaf extract, OLE, and AgNPs-OLE suggest that they possess significant anticancer activity against human breast cancer (T47D and MCF7) cell lines. These results prove that green synthesis of AgNPs via *O. europaea* extracts is an excellent way to create nanoparticles with well-defined sizes and morphologies, which could have various novel therapeutic applications.

This study focused on the green synthesis of AgNPs via medicinal plants as a fast, simple, economic, nontoxic, eco-friendly, and biocompatible technique. In addition, future optimization of the green synthesis conditions will help produce particles with well-defined sizes and morphologies, thereby improving the properties of the nanoparticles. This would enhance the widespread therapeutic application of AgNPs and would bring forward the investigation into toxicity and clinical research, offering good opportunities to fight several diseases and undesirable pathogens using nanotechnology with natural compounds, leading to new insights and new types of medicine.

## Figures and Tables

**Table 1:** Phytochemical analysis of *Olea europaea* extracts standard procedures.

Phytochemicals	Tests	References
**Phenolic**	**Ferric chloride test:** a few drops (3–4 drops) of ferric chloride solution were added to 1 ml of the plant extract. The formation of bluish black confirms the presence of phenols.	[20]

**Flavonoids**	**Lead acetate test:** a few drops of 10% dilute lead acetate solution were added to the plant extract. The formation of the yellow precipitate confirms the presence of flavonoids.	[21]

**Terpenoids**	**Liebermann–Burchard test:** a few drops of sulphuric acid were added to 1 ml of the plant extract, and the formation of a brown ring at the junction and red colour in the upper layer confirms the presence of terpenoids.	[22]

**Saponins**	**Frothing test:** plant extract was treated with distilled water in a tube and shaken well. The formation of stable froth confirms the presence of saponins.	[23]

**Table 2:** Phytochemical screening test results of *Olea europaea* extracts.

Tested phytochemicals	*Olea europaea leaf*	*Olea europaea* fruit
Phenolic	+	+
Flavonoids	+	+
Terpenoids	+	+
Saponins	+	−

+ presence and − absence.

## Data Availability

The data used to support the findings of this study are included within the article, and necessary explanations in relation to this can be obtained from the corresponding author upon request.
